# Preliminary fMRI findings in experimentally sleep-restricted adolescents engaged in a working memory task

**DOI:** 10.1186/1744-9081-5-9

**Published:** 2009-02-19

**Authors:** Dean W Beebe, Mark W DiFrancesco, Sarah J Tlustos, Kelly A McNally, Scott K Holland

**Affiliations:** 1 Division of Behavioral Medicine and Clinical Psychology, Cincinnati Children's Hospital Medical Center, 3333 Burnet Avenue, Cincinnati, Ohio, USA; 2 Department of Pediatrics, University of Cincinnati College of Medicine, 231 Albert Sabin Way, Cincinnati, Ohio, USA; 3 Department of Radiology, Cincinnati Children's Hospital Medical Center, 3333 Burnet Avenue, Cincinnati, Ohio, USA; 4 Psychology Department, University of Cincinnati College of Arts and Sciences, 2600 Clifton Avenue, Cincinnati, Ohio, USA

## Abstract

Here we report preliminary findings from a small-sample functional magnetic resonance imaging (fMRI) study of healthy adolescents who completed a working memory task in the context of a chronic sleep restriction experiment. Findings were consistent with those previously obtained on acutely sleep-deprived adults. Our data suggest that, when asked to maintain attention and burdened by chronic sleep restriction, the adolescent brain responds via compensatory mechanisms that accentuate the typical activation patterns of attention-relevant brain regions. Specifically, it appeared that regions that are normally active during an attention-demanding working memory task in the well-rested brain became even more active to maintain performance after chronic sleep restriction. In contrast, regions in which activity is normally suppressed during such a task in the well-rested brain showed even greater suppression to maintain performance after chronic sleep restriction. Although limited by the small sample, study results provide important evidence of feasibility, as well as guidance for future research into the functional neurological effects of chronic sleep restriction in general, the effects of sleep restriction in children and adolescents, and the neuroscience of attention and its disorders in children.

## Background

Inadequate sleep is endemic among adolescents in developed countries. Whereas clinical recommendations call for about 9 hours of nightly sleep in healthy adolescents [1], nearly half sleep less than 7 hours each school night [2]. Adolescent sleep restriction results in daytime inattention [3], but the neural mechanisms that underlie this effect remain unknown.

In adults, functional magnetic resonance imaging (fMRI) studies have shown that acute sleep deprivation can alter the activation patterns of two attention networks. In well-rested adults, the "*task positive*" network, which includes portions of the prefrontal cortex and posterior parietal lobes, shows *more *activity during a variety of tasks that require active allocation of attentional resources than during control tasks that require less mental engagement (e.g., "rest" periods or a simple perception task) [4,5]. In contrast, the "*task negative*" network, which includes the medial frontal lobes and posterior cingulate, is typically *less *active during attention-demanding tasks than control tasks [4,5]. Although some findings appear task-specific, when sleep-deprived adults perform normally on attention-demanding tasks, the divergent activation and deactivation patterns in task positive and task negative networks typically become even more pronounced [6-12], but when they perform poorly, this divergence is attenuated [6,7,12-16]. The former is believed to reflect a compensatory response to the burden of sleep deprivation on these networks, whereas the latter represents a breakdown in that response [6,8,11].

Although intriguing, these findings have been limited to adults, and may not generalize well to adolescents. Whereas adolescents often experience chronic partial sleep restriction (i.e., obtaining inadequate sleep across several consecutive nights), past fMRI studies have used 1- and 2-night complete sleep deprivation protocols. Moreover, adolescents are often asked to attend to tasks at a time much closer to their circadian nadir than adults because of early school start times and a delayed sleep phase [1]. Developmental shifts in the homeostatic sleep drive [17] may also affect adolescents' response to chronic sleep restriction.

We recently documented the feasibility and behavioral effects of experimental chronic sleep restriction in healthy adolescents [3]. Here we report pilot data on a subsample from that group who underwent fMRI examination while they completed a working memory task known to require effortful attention and to conform to the task-positive/task negative functional activation model.

## Methods

The experimental protocol and sample used for this study are detailed in Beebe et al. [3]. Briefly, healthy adolescents underwent a 3-week protocol which included a baseline week, followed in random order by a sleep restriction week (SR) and a healthy duration week (HD). Wake time, which did not significantly vary across the three weeks, was established as the time each subject would need to arise to attend an 8:30 am meeting. In contrast, bedtimes were systematically varied. Subjects self-selected their bedtimes during the baseline week, which was intended to stabilize subjects' sleep phase prior to any experimental manipulation. On the Monday-Friday nights of the SR week, subjects set a late bedtime that limited them to 6.5 hours in bed; on the Monday-Friday nights of the HD week, bedtime was set earlier to allow 10 hours in bed with lights out. Saturday and Sunday nights were considered "washout" periods, during which bedtime was subject-selected. Sleep was monitored via objective actigraphy and self-report. We intended to study 10 of the 20 subjects from the larger study, but obtained complete fMRI data on 6 due to equipment malfunction for 2 subjects, 1 falling asleep mid-scan, and 1 who misunderstood task instructions. These 6 subjects (mean age = 15.3 ± 0.7 years; 4 males) averaged 2 1/2 hours more sleep per night (range 1.7 – 3.0) in the HD condition than the SR condition (Table 1). On study-specific questionnaires [3], these subjects also self-reported much quicker sleep onset at night and greater difficulty waking in the morning, and parents reported observing greater daytime sleepiness and inattentive behaviors, during the SR week.

**Table 1:** Sleep and behavioral functioning across conditions

	**Baseline**	**Sleep restriction (SR)**	**Healthy duration (HD)**
**Actigraphy data**			
Duration (hr)***	7.7 ± 1.1	6.3 ± 0.5	8.7 ± 0.4
Efficiency*	89% ± 8	92% ± 6	85% ± 8
**Sleep diary data**			
Duration (hr)***	7.6 ± 1.0	6.4 ± 0.2	9.1 ± 0.3
Latency (min)**	12.6 ± 6.3	7.2 ± 6.4	32.5 ± 12.4
Difficulty waking scale*	5.2 ± 3.3	7.0 ± 2.8	2.4 ± 2.1
**Behavioral data per parent**			
Attention problems (raw)^†^	3.7 ± 2.5	5.7 ± 2.0	3.0 ± 1.9
Daytime sleepiness (raw)*	2.3 ± 1.5	4.3 ± 2.3	1.5 ± 1.6

^†^p < .10; *p < .05; **p < .005, ***p < .001 comparing the SR vs. HD sleep conditions. These findings are comparable to those shown in the full sample of the parent study. See Beebe et al. [3] for a full description of the parent study, including sleep and behavioral measures.

Between 9 and 11:30 a.m. on the Saturday mornings at the end of each experimental week, subjects completed a computerized n-back task while undergoing fMRI monitoring. We chose an n-back task because such tasks allow for strong discrimination between basic perceptual/motor demands versus higher-level demands placed on attention and working memory, resulting in well-established functional activation patterns that conform to the task-positive versus task negative network model [18]. In the n-back task we used [19], subjects viewed numbers from 1 to 4 that consistently appeared in one of four visual quadrants. In the "0-back" control task, subjects were to press a button to match each stimulus as it was presented. In the "2-back" task, subjects indicated the stimulus that appeared two steps earlier. Stimuli were presented in a periodic block-design paradigm in which 30 second intervals of the two conditions were interleaved beginning with a block of the control task followed by 5 blocks of each task. Within each block, 17 stimuli appeared (ISI = 1.7 sec, 0.2 second blank screen between stimuli); preceding each block there was a 3 second visual warning of the change in task (total task duration: 6 min, 3 sec).

Scans were performed on a Bruker Biospec 30/60 3T MRI scanner (Bruker Biospin, Ettlingen, Germany) (n = 4) and on a Siemens Trio 3T scanner (Siemens AG, Munich, Germany) (n = 2). Activation patterns and n-back performance were very similar across scanners, so data were pooled for group analyses. Both scanners used a T2*-weighted, gradient-echo, echo planar imaging sequence for the fMRI scans (TR = 3000 ms, TE = 38 ms, FOV = 25.6 × 25.6 cm, matrix = 64 × 64, 25 5-mm axial slices). A high-resolution, T1-weighted, inversion-prepared MDEFT whole brain scan [20,21] served as anatomical reference for coregistration and overlay of functional data (TR = 16.5, TE = 8, inversion recovery time 550 ms, FOV = 25.6 × 19.2 × 19.2 cm, matrix = 256 × 128 × 128). Preprocessing of each session included removal of imaging artifacts. The initial 10 images were discarded to ensure T1 relaxation equilibrium. Spatial preprocessing using SPM5 [22] included motion correction by realignment, normalization to the MNI brain reference space [23], and spatial filtering with an 8 mm Gaussian kernel. Signal intensity was globally normalized for each functional image volume. Analysis of functional data began with a voxel by voxel assessment of contrast between the 2-back and the 0-back tasks under the general linear model [24], regressing against the n-back block time course with realignment parameters used as covariates. A group composite T-score map was then constructed for each sleep condition by random effects analysis [25] and overlaid on the subjects' average anatomical reference image.

The small sample did not afford enough statistical power to make voxel-by-voxel comparisons across conditions. Instead, we took a region of interest (ROI) approach, using automated masks [26] to define two bilateral ROIs: a *task positive *ROI comprised of the dorsolateral prefrontal cortex and the inferior parietal lobes, and a *task negative *ROI comprised of the medial frontal gyri and posterior cingulate. Within each subject and condition, an activation score for the *task positive *ROI was calculated as the mean positive T-score for voxels in the ROI, and a deactivation score for the *task negative *ROI was calculated as the mean negative T-score for voxels in that area. We then applied a bootstrapping procedure for each ROI in each condition by resampling the voxel T values 1000 times (with replacement) to estimate the sampling distribution of the ROI scores, thereby allowing us to examine the possibility that differences were due to sampling error.

## Results

**Figure 1:**
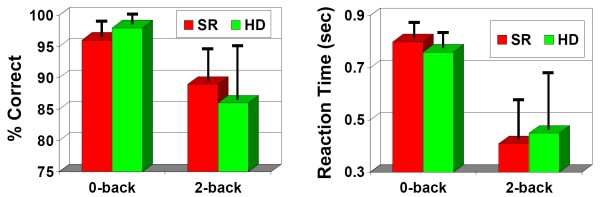
**Accuracy and reaction time were comparable across sleep conditions on the 0-back and 2-back tasks (*p *> .10)**. SR = Sleep Restriction, HD = Healthy Sleep Duration.

**Figure 2:**
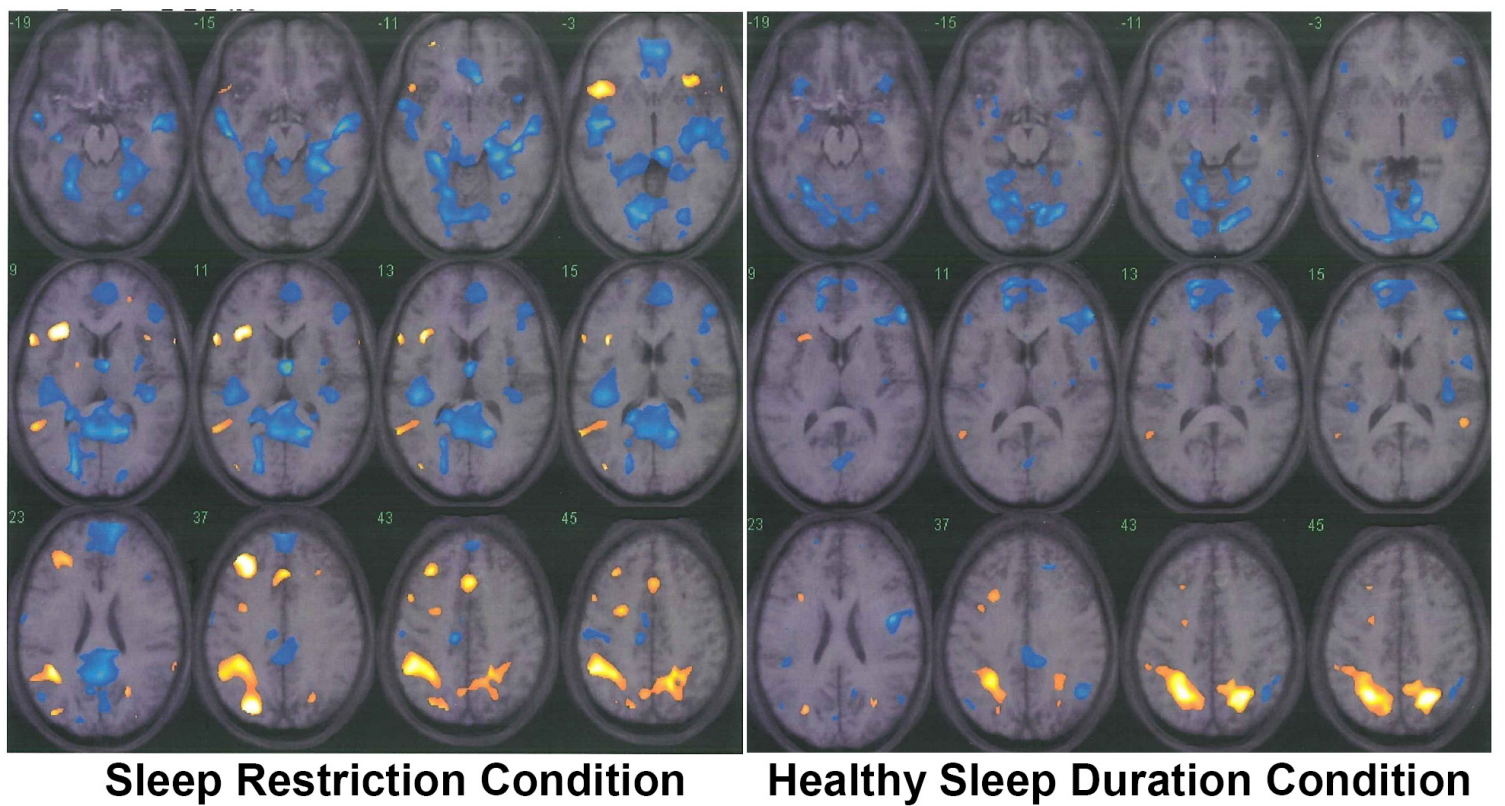
**Composite activation/deactivation maps, showing contrast of 2-back task with 0-back task in each experimental sleep condition**. Warm colors (orange to yellow) reflect voxels that are more active during 2-back than the control task, with a threshold of T > 3; Cool colors (blue) reflect relative deactivation during 2-back, T < -3. Slices shown are at -19, -15, -11, -3, +9, +11, +13, +15, +23, +37, +43, and +45 mm.

**Figure 3:**
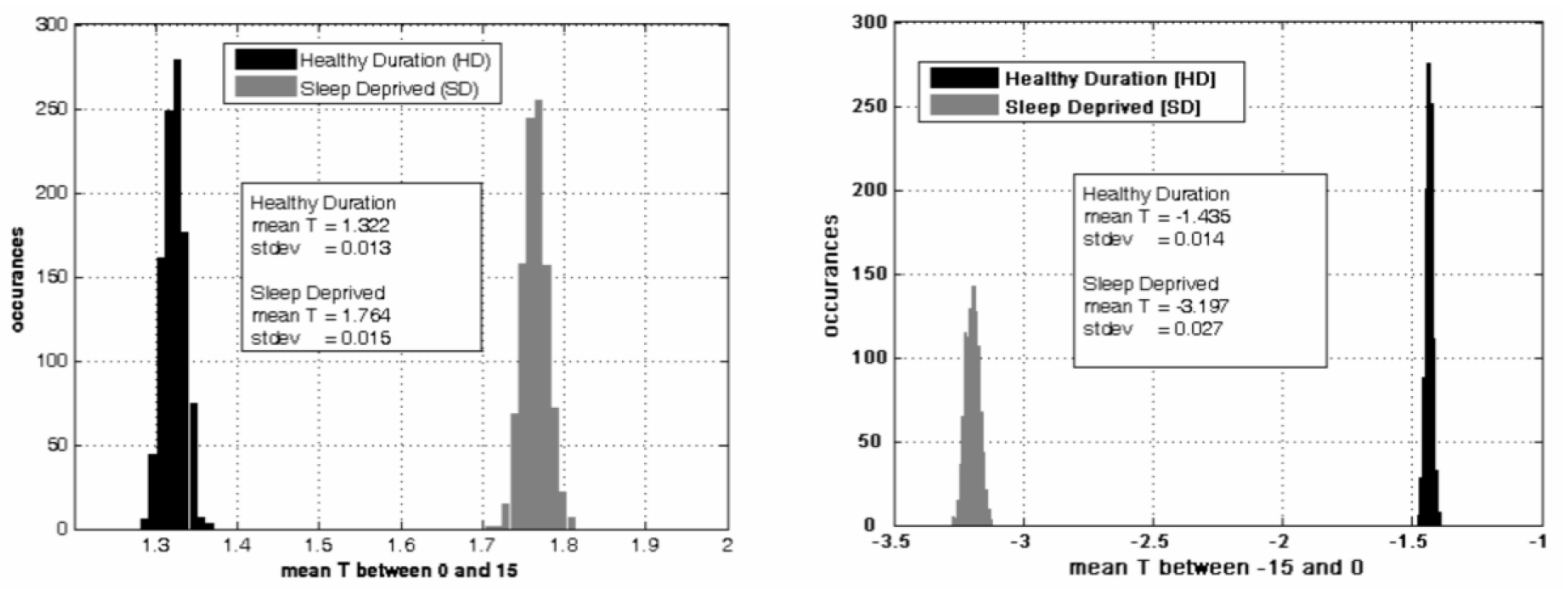
**Histograms reflecting results of the bootstrap resampling procedure across experimental sleep conditions**. The panel on the left illustrates the greater activation in the task positive ROI during the Sleep Restriction (SR) condition than during the Healthy Sleep Duration (HD) condition. The panel on the right illustrates the greater deactivation in the task negative ROI during the SR condition than during the HD condition.

N-back performance was comparable across the two experimental conditions (Figure 1). Consistent with prior studies (e.g., [18,19]), in both conditions the 2-back task resulted in relative activation in task positive regions and deactivation in task negative regions. These are graphically illustrated in Figure 2 and indexed in Tables 2 and 3 by conventional label, Brodmann's area [27] and Talairach coordinates to serve as a baseline reference. Though the activation patterns were similar across conditions, the *intensity *of such activation/deactivation differed across conditions. Relative to HD, in the SR condition subjects showed greater activation in the task positive ROI and more deactivation in the task negative ROI (Figure 3). The estimated sampling distributions did not overlap across conditions, suggesting that the mean differences were not due to sampling error.

**Table 2:** Brain regions activated under the n-back task for the sleep restriction (SR) and healthy duration (HD) conditions

		**SR**		**HD**	
**Brain region**	**Brodmann areas**	**Centroid location**	**Volume [cc]**	**Centroid location**	**Volume [cc]**
Precentral gyrus	9			L, -33, 10, 30	1.4
Precuneus	7, 19	L, -27, -77, 35 L, -15, -68, 50 R, 18, -62, 48	1.8 0.6 0.7	R, 21, -64, 45	3.0
Superior parietal lobule	7			L, -23, -60, 44	5.5
Middle frontal gyrus	6	L, -24, 0, 53	2.1		
Dorsolateral prefrontal gyrus	9	L, -32, 31, 34	2.8		
Insula	13	L, -41, 18, 3 R, 31, 25, -1	3.8 0.8		
Inferior parietal lobule	40	L, -41, -49, 37	3.8		
Cingulate gyrus	32	B, 1, 20, 39	0.6		

L = left hemisphere, R = right hemisphere, B = bilateral. Clusters satisfy a significance threshold of p < 0.005 (uncorrected) and a volume threshold of 0.5 cc. Centroid locations are in Talairach coordinates.

**Table 3:** Brain regions deactivated under the n-back task for the sleep restriction (SR) and healthy duration (HD) conditions

		**SR**		**HD**	
**Brain regions**	**Brodmann areas**	**Centroid location**	**Volume [cc]**	**Centroid location**	**Volume [cc]**
Precentral gyrus	6			R, 51, -2, 21	0.9
Postcentral gyrus	3	L, -33, -31, 59	1.0		
Inferior parietal lobule	40			R, 47, -59, 36	1.0
Inferior frontal gyrus	46			R. 49, 35, 8	1.7
Lingual gyrus, vermis	17	R, 24, -65, -17 R, 23, -87, 2	0.5 1.5	B, 1, -74, -8	16.7
Cingulate gyrus	31			R, 10, -34, 35	0.6
Anterior cingulated gyrus, medial frontal gyrus	32, 10	B, 4, 47, 8	9.8	L, -13, 55, 12	1.6
Posterior cingulate gyrus	23, 29, 30	B, -6, -56, 6	30.7		
Parahippocampal gyrus, fusiform gyrus	35, 36, 37	R, 38, -25, -10	12.2		
Superior temporal gyrus	22	L, -47, -18, 1	7.0		
Middle temporal gyrus	21	L, -51, -11, -16	0.7		

L = left hemisphere, R = right hemisphere, B = bilateral. Clusters satisfy a significance threshold of p < 0.005 (uncorrected) and a volume threshold of 0.5 cc. Centroid locations are in Talairach coordinates.

## Discussion

As the first published imaging study of the functional neural consequences of sleep restriction in a pediatric sample, this study provides important evidence of feasibility and preliminary findings in this understudied population. The adolescents in this study overall appeared resilient to chronic sleep restriction in their n-back performance, but their neural activation differed across conditions. Adolescents showed greater relative activation of the task positive attention network and deactivation in the task negative network after chronic sleep restriction than when well-rested. A compensatory cerebral response appeared to be elicited during sleep restriction [11] that may have helped focus attention and suppressed extraneous mental processes [5], resulting in relatively preserved performance. This is consistent with prior studies of adults, in which sleep-deprived subjects who maintain their performance on tasks that require concentrated effort show particularly heightened activation in the task-positive attention network, particularly low activation in the task-negative attention network, or both [6-12].

Future research will be needed to determine whether or how this compensatory response breaks down, as adolescents who have experienced chronic sleep restriction ultimately show reduced attention in applied settings [3]. Based upon findings from prior adult research, one might expect a correspondence between task failure and attenuation or loss of activation in the task-positive network, relative activation within the task-negative network, or both [6,7,12-16]. Such an effect may not be unique to the sleep deprived state, as studies of subjects who have not been intentionally deprived of sleep have shown that task-negative regions are relatively more active when adults subjectively report daydreaming [28] or objectively display momentary lapses in attention [29].

This study also illustrates the utility of a protocol that could advance research into the neuroscience of pediatric attention and its disorders. The most common approach to understanding neurological aspects of attention in children has been to compare those with known deficits to healthy controls, which carries potential confounds that can be difficult to disentangle. Our within-subjects experimental method could provide a less confounded tool for understanding attention network functioning under healthy and sub-optimal conditions.

Finally, present findings demonstrate that it is feasible to examine functional activation patterns after *chronic *sleep *restriction*. To our knowledge, such research has not been previously published, despite the ubiquity of chronic sleep restriction among adolescents and its frequent occurrence in adults [30]. Present results are consistent with the hypothesis that the cumulative effects of chronic sleep restriction are similar to those seen after acute sleep deprivation, but more research is needed to verify and elaborate upon our findings. Of note, it is not known how changes in functional activation unfold over the course of chronic sleep restriction; recent quantitative EEG results suggest region-specific, nonlinear changes in neural activity over the course of a week of restricted sleep in healthy adults [31].

## Limitations

Findings from this study are considered preliminary because of several limitations, including the reliance on a single working memory task, the use of multiple scanners, and a fixed sleep protocol across all subjects. More importantly, the small sample precluded voxel-wise comparisons across conditions and diminished statistical power to detect effects on n-back accuracy or reaction time. Nevertheless, the within-subjects experimental design produced robust ROI-based findings and reduced possible confounds. If replicated, these findings have the potential to guide targeted palliative treatments for adolescents with refractory sleep disorders or sleep problems that are secondary to medical illness. Our findings also set the stage for future research investigating questions of reversible versus irreversible effects of chronic sleep restriction during brain development.

## Conclusion

Adolescents in our sample displayed a cerebral response to chronic sleep restriction that appeared similar to that described in acutely sleep-deprived adults. Although present findings have limitations, they provide important evidence of feasibility and guidance for novel lines of research into the functional neurological effects of chronic sleep restriction, the effects of pediatric sleep restriction, and the neuroscience of attention in children.

## Abbreviations

FOV: Field of View; fMRI: functional Magnetic Resonance Imaging; HD: Healthy Duration (condition requiring 10 hours in bed Monday-Friday nights); ISI: Interstimulus Interval; SR: Sleep Restriction (condition limiting time in bed to 6.5 hours Monday-Friday nights); SPM: Statistical Parametric Mapping software; TE: Echo Time; TR: Repetition Time; ROI: Region of Interest; MDEFT: Modified Driven Equilibrium Fourier Transform; MNI: Montreal Neurological Institute; SPM5: Statistical Parametric Mapping software.

## Competing interests

The authors declare that they have no competing interests.

## Authors' contributions

DWB oversaw the project, directed the experimental sleep manipulation, monitored subject adherence to the sleep manipulation, conducted the behavioral performance analyses, and drafted the manuscript. MWD and SKH oversaw the collection of anatomical and functional imaging data. MWD also conducted primary imaging analyses, drafted portions of the manuscript, and had conceptual input in the research design and interpretation of findings. SJT and KAM assisted in data collection and contributed to manuscript revisions. SKH oversaw the imaging analyses, had conceptual input in the research design, and contributed to manuscript revisions. All authors read and approved the final manuscript.

## References

[B1] Carskadon MA (2002). Adolescent sleep patterns: Biological, social, and psychological influences.

[B2] Wolfson AR, Carskadon MA (1998). Sleep schedules and daytime functioning in adolescents. Child Dev.

[B3] Beebe DW, Fallone G, Godiwala N, Flanigan M, Martin D, Schaffner L, Amin R (2008). Feasibility and behavioral effects of an at-home multi-night sleep restriction protocol for adolescents. Jn Child Psychol Psychiatry.

[B4] Fox MD, Snyder AZ, Vincent JL, Corbetta M, Van Essen DC, Raichle ME (2005). The human brain is intrinsically organized into dynamic, anticorrelated functional networks. Proc Natl Acad Sci USA.

[B5] Sonuga-Barke EJ, Castellanos FX (2007). Spontaneous attentional fluctuations in impaired states and pathological conditions: a neurobiological hypothesis. Neurosci Biobehav Rev.

[B6] Choo WC, Lee WW, Venkatraman V, Sheu FS, Chee MW (2005). Dissociation of cortical regions modulated by both working memory load and sleep deprivation and by sleep deprivation alone. Neuroimage.

[B7] Thomas RJ, Kwong K (2006). Modafinil activates cortical and subcortical sites in the sleep-deprived state. Sleep.

[B8] Drummond SP, Brown GG, Salamat JS, Gillin JC (2004). Increasing task difficulty facilitates the cerebral compensatory response to total sleep deprivation. Sleep.

[B9] Drummond SP, Brown GG, Stricker JL, Buxton RB, Wong EC, Gillin JC (1999). Sleep deprivation-induced reduction in cortical functional response to serial subtraction. Neuroreport.

[B10] Drummond SP, Gillin JC, Brown GG (2001). Increased cerebral response during a divided attention task following sleep deprivation. J Sleep Res.

[B11] Drummond SP, Meloy MJ, Yanagi MA, Orff HJ, Brown GG (2005). Compensatory recruitment after sleep deprivation and the relationship with performance. Psychiatry Res.

[B12] Lim J, Choo WC, Chee MW (2007). Reproducibility of changes in behaviour and fMRI activation associated with sleep deprivation in a working memory task. Sleep.

[B13] Chee MW, Choo WC (2004). Functional imaging of working memory after 24 hr of total sleep deprivation. J Neurosci.

[B14] Habeck C, Rakitin BC, Moeller J, Scarmeas N, Zarahn E, Brown T, Stern Y (2004). An event-related fMRI study of the neurobehavioral impact of sleep deprivation on performance of a delayed-match-to-sample task. Brain Res Cogn Brain Res.

[B15] Chee MW, Chuah LY, Venkatraman V, Chan WY, Philip P, Dinges DF (2006). Functional imaging of working memory following normal sleep and after 24 and 35 h of sleep deprivation: Correlations of fronto-parietal activation with performance. Neuroimage.

[B16] Drummond SP, Bischoff-Grethe A, Dinges DF, Ayalon L, Mednick SC, Meloy MJ (2005). The neural basis of the psychomotor vigilance task. Sleep.

[B17] Jenni OG, Achermann P, Carskadon MA (2005). Homeostatic sleep regulation in adolescents. Sleep.

[B18] Cabeza R, Nyberg L (2000). Imaging cognition II: An empirical review of 275 PET and fMRI studies. J Cogn Neurosci.

[B19] Adler CM, Delbello MP, Mills NP, Schmithorst V, Holland S, Strakowski SM (2005). Comorbid ADHD is associated with altered patterns of neuronal activation in adolescents with bipolar disorder performing a simple attention task. Bipolar Disord.

[B20] Duewell S, Wolff SD, Wen H, Balaban RS, Jezzard P (1996). MR imaging contrast in human brain tissue: assessment and optimization at 4 T. Radiology.

[B21] Ugurbil K, Garwood M, Ellermann J, Hendrich K, Hinke R, Hu X, Kim SG, Menon R, Merkle H, Ogawa S (1993). Imaging at high magnetic fields: initial experiences at 4 T. Magn Reson Q.

[B22] Statistical Parametric Mapping. http://www.fil.ion.ucl.ac.uk/spm/.

[B23] Evans AC, Collins DL, Mills SR, Brown ED, Kelly RL, Peters TM (1993). 3D statistical neuroanatomical models from 305 MRI volumes. Proc IEEE Nucl Sci Symp Med Imaging.

[B24] Worsley KJ, Liao CH, Aston J, Petre V, Duncan GH, Morales F, Evans AC (2002). A general statistical analysis for fMRI data. NeuroImage.

[B25] Friston KJ, Holmes AP, Price CJ, Buchel C, Worsley KJ (1999). Multisubject fMRI studies and conjunction analyses. NeuroImage.

[B26] Wake Forest University School of Medicine Advanced Neuroscience Imaging Research. http://www.fmri.wfubmc.edu/download.htm.

[B27] Brodmann K, Garey L (1999). Brodmann's "Localisation in the cerebral cortex".

[B28] Mason MF, Norton MI, Van Horn JD, Wegner DM, Grafton ST, Macrae CN (2007). Wandering minds: the default network and stimulus-independent thought. Science.

[B29] Weissman DH, Roberts KC, Visscher KM, Woldorff MG (2006). The neural bases of momentary lapses in attention. Nat Neurosci.

[B30] Institute of Medicine Committee on Sleep Medicine and Research (2006). Sleep disorders and sleep deprivation: An unmet public health problem.

[B31] Cote KA, Milner CE, Osip SL, Baker ML, Cuthbert BP (2008). Physiological arousal and attention during a week of continuous sleep restriction. Physiol Behav.

